# *Ganoderma tuberculosum* Liquid Culture With Vineyard Pruning Extracts for Bioactive Composite Production With Antiproliferative Activity

**DOI:** 10.1155/2024/5245451

**Published:** 2024-10-24

**Authors:** Lucia T. Angulo-Sanchez, María C. Cruz-Félix, Max Vidal-Gutiérrez, Heriberto Torres-Moreno, Óscar A. Muñoz-Bernal, Emilio Álvarez-Parrilla, Ramón E. Robles-Zepeda, Osiris Álvarez-Bajo, Aldo Gutiérrez, Martín Esqueda

**Affiliations:** ^1^Centro de Investigación en Alimentación y Desarrollo, A.C. Carretera Gustavo Enrique Astiazarán Rosas 46, La Victoria, Hermosillo CP. 83304, Sonora, Mexico; ^2^Universidad de Sonora, Campus Navojoa, Departamento de Ciencias Químico, Biológicas y Agropecuarias, Lázaro Cárdenas del Río 100, Francisco Villa, Navojoa CP. 85880, Sonora, Mexico; ^3^Universidad de Sonora, Campus Caborca, Departamento de Ciencias Químico, Biológicas y Agropecuarias, Avenida K SN, Eleazar Ortiz, H. Caborca CP. 83600, Sonora, Mexico; ^4^Universidad Autónoma de Ciudad Juárez, Instituto de Ciencias Biomédicas, Av. Benjamín Franklin 4650, Condominio La Plata, Ciudad Juárez CP. 32310, Chihuahua, Mexico; ^5^Universidad de Sonora, Campus Hermosillo, Departamento de Ciencias Químico Biológicas, Blvd. Luis Donaldo Colosio y Rosales s/n, Centro, Hermosillo CP. 83000, Sonora, Mexico; ^6^Consejo Nacional de Ciencia y Tecnología-Universidad de Sonora, Blvd. Luis Encinas y Rosales s/n, Hermosillo CP. 83000, Sonora, Mexico

## Abstract

*Ganoderma* species have been studied for their pharmacological approaches, such as anticancer, antitumor, antiproliferative, and antioxidant activity. Elicitors are used to increase *Ganoderma* bioactive composite production. This study aims to evaluate the antiproliferative activity of ethanolic extracts from mycelium of *Ganoderma tuberculosum (G. tuberculosum)* grown in a liquid medium with vineyard pruning waste (VPW) extracts as elicitors. Ethanolic and aqueous VPW extracts contain resveratrol dimer 4, resveratrol tetramer 1, and naringenin, while toluene and chloroform extracts contain tetradecanoic acid, hexadecanoic acid, and octadecanoic acid. Polar and nonpolar extracts could be promising elicitors for increasing bioactive molecules. Catechin gallate showed the highest correlation (*r* = 0.66) with biomass. Mycelial ethanolic extracts of *G. tuberculosum* (native strain from the Sonoran Desert) and *Ganoderma lucidum* (*G. lucidum*) (control) were analyzed by ESI-IT-MS, and 27 molecules were identified for the two species. They showed antiproliferative activity against the A549 and C-33 A cell lines but not for ARPE-19. *G. tuberculosum* culture with VPW had quinic acid, ganodermenonol, ganoderic acid I (GA-I), C2 (GA-C2), and 20-hydroxylucidenic acid P, among others. Molecular docking of ganodermenonol, GA-I, and GA-C2 demonstrates significant interaction with tumor necrotic factor (TNF-*α*). These ethanolic extracts of *Ganoderma* are promising sources of bioactive triterpenoids. Their antiproliferative activity did not change between species or treatment. Likewise, the *G. tuberculosum* and *G. lucidum* extracts only affected cancer cell lines. This property seems promising for pharmacological applications of these fungal extracts.

## 1. Introduction

*Ganoderma* species have been used for medical purposes for 6000 years; its consumption has been related to prolonged life, alleviated aging, and relaxed body worldwide [1]. Studies of *Ganoderma* species have been developed to identify their bioactive composites such as antiproliferative [2], antitumoral [3], anti-inflammatory [4, 5], and antioxidant activity [6], among others. Around 400 composites and > 150 triterpenoids of *Ganoderma* spp., fruiting bodies, spores, and mycelia have been reported [7]. However, the native strains of *Ganoderma* from deserts have been poorly studied, such as *Ganoderma tuberculosum* (*G. tuberculosum*), a strain collected in a cactus [8]. Research continues to identify, discover, and characterize triterpenoids with pharmacological properties from other *Ganoderma* species and improve the culture conditions.

Most of the described composites come from wild or cultivated fruiting bodies, and the minority are from mycelia grown in liquid medium. Growing conditions such as substrate are known to change the chemical and physical properties of *Ganoderma* species [9]. G*anoderma* spp. have been used as a factory for molecules such as triterpenoids, which is better than synthesizing these composites due to their complex structure [10]. A novel strategy to enhance triterpenoid production is the liquid culture using elicitors [11], different culture conditions [12], sources [13], and techniques. One of them is to limit the source of nitrogen, the quantity of glucose, or the administration of oxygen to promote ganoderic acid (GA) production [14]. Also, the pH could affect the triterpenoids and biomass production, with 5.5 being the best for cultivation [15]. Xu et al. [13] developed an optimal medium, including corn flour and soybean powder, to improve the GA's synthesis, but elicitor concentration is crucial because an opposite effect can occur [16]. Some phenols are used as a biological control against *Ganoderma* because they reduce cell viability [17] and can inhibit some enzymes [18]. One strategy to enhance the production of composites and biomass that has been studied could be using agrowastes to improve liquid culture and economic value.

Agrowaste is important in mushroom cultivation due to an increased by-product value and biotransformation [19]. Sonora, Mexico, has the highest grape production with 326,849 t, 16.15 udm/ha of yield in 2022, which enhances vineyard pruning wastes (VPWs) [20]. One way to approach this waste without burning it and contaminating the environment is to use it as a source of lignocellulose for growing mushrooms [21]. In addition, VPW has been used as a substrate in the solid culture of *Ganoderma* spp. to increase the fruiting bodies' production [22]. On the other hand, Harris-Valle et al. [23] reported that polar extracts of VPW improve mycelial output and increase the activity of ligninolytic enzymes in *Lentinula edodes* (*L. edodes*) mycelial culture, but they do not characterize the extracts.

This study aims to evaluate the potential of VPWs to improve the metabolism of *G. tuberculosum* (Sonoran Desert native strain) and *Ganoderma lucidum* (*G. lucidum*) (control). VPW enhanced biomass production in liquid culture [24] without evidence of GA's biosynthesis and its antiproliferative activity against the cancer cell lines (HeLa, C-33, and A549).

## 2. Materials and Methods

### 2.1. VPW Extracts

The sun-dried VPW was collected in La Costa de Hermosillo, Sonora, Mexico (29°01′1″N, 111°34′24″W). 120 g of VPW was pulverized into particles of 1-2 mm. 30 g sacks with VPW were placed into the Soxhlet chamber; the extraction procedure was performed by toluene, chloroform, ethanol, and water due to their polarity with 250 mL by solvent for 3 h four times each. We used the boiling point reference for each solvent. Organic solvents were dried in bathwater (50°C) and lyophilized in the aqueous part. Dry extracts were weighed to determine the yield percentage and storage at room temperature (25°C) in a desiccator. For liquid culture treatment, the dry extracts were dissolved in 70% ethanol at the concentration of 1 g/100 mL separately, and then polar and nonpolar extracts were combined and stored at 4°C [23].

#### 2.1.1. Phenolic Profile of VPW

The phenolic profile of VPW was determined in an Agilent Infinity Series 1290 LC system combined with an Agilent 6500 Series Q-TOF LC/MS system (Agilent Technologies, Santa Clara, California, United States of America) and applying an Agilent mass hunter software MS (Agilent Technologies, Santa Clara, California, United States of America), according to the protocol of Muñoz-Bernal et al. [25]. The high-performance liquid chromatography (HPLC) system was a 1290 Infinity quaternary pump with a built-in degasser, a 1290 autosampler with temperature control, a 1290 Infinity thermostat column compartment, and a 1290 Infinity diode-array detector. The column used was Zorbax Eclipse plus C18 (50 × 2.1 mm, 1.8 *μ*m) (Agilent Technologies, Santa Clara, California, United States of America) at 25°C. A multistep gradient method was used for the separations where formic acid was 0.01% in water (A) and acetonitrile 100% (B). The elution program was 0-1 min 10% B, 1–4 min 30% B, 4–6 min 38% B, 6–8 min 60% B, 8–8.5 min 60% B, and 8.5–9 min 10% B. The extracts of toluene and chloroform were diluted in 100 *μ*L of toluene and chloroform (ACS grade), respectively. Then, 900 *μ*L of acetonitrile (HPLC–mass spectrometry grade) was added. Ethanol and water extracts were diluted in acetonitrile/methanol (50:50 v/v). The final concentration of all samples was 3 mg/mL, filtered through a 0.45 *μ*m nylon syringe filter, and poured into HPLC vials; the injection volume was 3 *μ*L.

The Agilent 6530 accurate mass Q-TOF LC/MS was in negative mode equipped with an electrospray ion (ESI) source. Drying gas was used; nitrogen at 340°C, with a flow rate of 13 L/min, 60 psi of pressure of the nebulizer, capillary voltage 175 V, and 100–1100 m/z of scanning mass to charge range of the Q-TOF mass analyzer, while for MS/MS, scanning were from 100 to 1000 m/z. Phenolic compound identification was performed using retention times (RT) and mass spectra obtained from Q-TOF-MS using Mass Hunter Qualitative software version B.07.00. The molecular feature extraction (MFE) tool was used to identify the coeluted compounds, and the generate formula (GF) tool was used to obtain the high-resolution isotopic distribution and exact mass (±5 ppm in contrast with the theoretical mass). MS/MS fragments were compared to the METLIN database in the mass hunted PCDL manager for metabolomics B.07.00. The match score was > 75% to identify compounds, and lower compounds were taken as unidentified compounds [26]. Spectral information from all the identified compounds is presented in Tables S1 and S2.

#### 2.1.2. Lipid Profile of Nonpolar Extracts of VPW

The lipid profile in GC-MS was for the nonpolar extracts of VPW: toluene and chloroform. These analyses were performed in a gas chromatograph coupled to a mass spectrophotometer, GCMS-QP2010 ultra (Shimadzu). The capillary column was ZB-5MSi (30m × 0.25 mm ID X 0.2 *μ*m). Helium gas was the mobile phase, and the procedure was 100°C holding for 5 min, the rate of 4°C/min at 250°C holding for 15 min. The conditions were source T° at 200°C, the interface was 250°C, EI was 70 eV, and the AMU was 35–500. The injection temperature was 250°C; the samples were derivatized with 100 *μ*L of N,O-*bis*(trimethylsilyl)trifluoroacetamide and incubated at 60°C for 60 min in sonication. The samples were prepared at 1 mg/mL, filtered with TITAN filters, and injected 1 *μ*L for each extract. Each peak was searched in NIST11.lib, considering matches with a major 70% identity [27].

### 2.2. Fungal Biomass

The control strain of *G. lucidum* (Curtis) P. Karst. (34D) was purchased from Fungi Perfecti, while the native strain from the Sonoran Desert, *G. tuberculosum* Murrill (BH-17), was provided by the Fungus Collection of the Plant and Fungi Biotechnology Laboratory of the Research Center in Food and Development. *G*. *tuberculosum* was previously cited as *Ganoderma oerstedii* (Fr.) Murrill [8] and was collected in the Sonoran Desert, locality of Pueblo de Alamos, Sonora, Mexico (29°11′11.97″N, 110°8′48.57″W), growing on a cactus *Stenocereus thurberi* (Engelm.) Buxb., being the first report of *Ganoderma* associated with a cactus [8]. Phylogenetic affinities of strain (BH-17) were inferred based on multilocus (ITS rDNA, *rpb2*, and *tef1-α*) DNA sequences (data not shown). The strain maintenance was in malt extract agar (MEA) at 28°C in darkness. The mycelial biomass was grown in a 1 L flask with 400 mL of the optimal medium at 25°C on a shaker (LAB LINE) at 120 rpm and 25°C in darkness. The culture medium was composed of corn flour (21 g), protein powder (7 g), sucrose (16 g), and peptone (2.93 g) [13]. In addition, the medium was supplemented with ethanol for the control (C) and VPW extracts (T) formulation. The treatment of VPM (T) was prepared in 70% ethanol. *G. tuberculosum* was with 500 *μ*g/mL of VPW polar extracts (ethanol:water, 1:1), while *G. lucidum* 1000 *μ*g/mL of all extracts (toluene:chloroform:ethanol:water, 1:1:1:1) VPW extract. In a previous study [24], the VPW extract concentration for higher biomass production per species was determined by the response surface analysis. The inoculum consisted of eight mycelial discs (1 cm diameter) for each flask. The culture was collected at 12 and 15 days by filtration and stored at −20°C to lyophilize. The lyophilization program conditions were vacuum and freeze for 30 min, vacuum and freeze for 1 h, drying at 32°C for 30 min, and drying at 32°C for 46 h. The biomass was weighed to measure its yield.

### 2.3. Fungal Biomass Extract

An ethanolic extraction was carried out from the biomass of *G. lucidum* and *G. tuberculosum* to obtain triterpenoids. The proportion for the extraction was 1:10, where 1 g of lyophilized and macerated biomass and 10 mL of ethanol samples were sonicated for 30 min (Ultra Sonic Cleaner, Branson 2210R-MT), with a power of 210.6 W and operating frequency of 40 kHz at the beginning and finalized the 7 days. The extract was collected by centrifugation and dried at 25°C.

### 2.4. Antiproliferative Activity

The antiproliferative activity of *Ganoderma* extracts was evaluated by MTT assay on HeLa (human cervical adenocarcinoma), C-33 A (human cervical carcinoma), A549 (non–small-cell lung cancer cells), and ARPE-19 (noncancerous retinal pigment epithelium) cell lines. Briefly, a cell suspension (200,000 cells/mL) was placed in a 96-well plate (Costar, Corning, NY, USA). The extracts were dried with a rotavap and prepared with DMSO at 25% of the final concentration. The cells were incubated for 24 h, and then 50 *μ*L of the extract (25–200 *μ*g/mL) was added to each well and incubated for 48 h. After removing the culture medium, the well was washed with PBS 1X, and 100 *μ*L of MTT solution (5 mg/mL) were added. Then, it was incubated for 4 h, and 100 *μ*L of acidic isopropanol was added to dissolve the formazan crystals. After 10 min in darkness, the absorbance was read at 630 and 570 nm wavelengths in a microplate reader (iMark microplate absorbance reader, Bio-Rad, Lab., Mexico). The antiproliferative activity was determined as IC_50_ value using Excel Microsoft Office 365. The morphological changes induced by the extracts at 24 h after the treatment were analyzed with microscopy inverted at 40x magnification [28].

### 2.5. Ion Trap Mass Spectrometry Assay

Samples at a concentration of 10 ppm were analyzed on a Thermo Scientific LTQ XL mass spectrometer with an ion tramp linear analyzer equipped with an electrospray ionization source (ESI-IT-MS/MS) and atmospheric pressure chemical ionization (APCI-IT-MS/MS) (Thermo, San Jose, California, United States of America). For ESI-IT-MS/MS in the negative mode, a flow of 10 *μ*L/min, tube lens at −200 V, capillary temperature at 280°C, −40 V voltage in the capillary, 6.0 kV in source, and a flow of 45 arbitrary units of enveloping gas were used. For the positive mode, a flow rate of 10 *μ*L/min, 150 V tube lens, 280°C capillary temperature, 35 V in capillary, 6.0 kV in source, and the flow of 45 arbitrary units of sheath gas were used. For both modes, a collision energy of 30 eV was used for multistay fragmentation using a collision-induced dissociation (CID) method with helium for ionic activation. For APCI-IT-MS/MS, the negative mode, a flow of 10 *μ*L/min, tube lens at −100 V, capillary temperature at 280°C, −37 V voltage in the capillary, 1.6 kV in source, and a flow of 30 arbitrary units of enveloping gas were used. For the positive mode, a flow rate of 10 *μ*L/min, 125 V tube lens, 280°C capillary temperature, 9 V in capillary, 1.6 kV in source, and the flow of 30 arbitrary units of sheath gas were used [29].

### 2.6. Compound-Target Prediction

The GAs identified by ESI-IT-MS were used to predict potential targets. The GAs analogs and their SMILES files were retrieved from the PubChem database (https://pubchem.ncbi.nlm.nih.gov/). Then, using the SWISS target prediction online server (https://www.swisstargetprediction.ch/), we submit the SMILES to search for the possible target. The best target was chosen based on probability < 1, as mentioned in the results. We select the best target with > 0.5 but < 1 for molecular docking [30].

### 2.7. Molecular Docking

The best target was selected in the protein data bank (https://www.rcsb.org/). For 3D molecules, we search in PubChem for the files (.sdf), visualize, and convert to mol2 format in USCF Chimera [31]. When the composite was not in the database, we drew it in the MolView online program (https://molview.org/) and converted it to mol2 format. For molecular docking, we used the online server SWISSdock (https://www.swissdock.ch/) [32]. The results were visualized in USCF Chimera, and the variation that showed an interaction was selected. Then, to visualize the possible interactions between GA and protein TNF-*α* (pdb: 2az5), the Ligplot program was used [33], and to take images, the program PyMOL (https://pymol.org/) [34].

### 2.8. Statistical Analysis

The phenolic and lipid profiles were submitted to a point-biserial correlation, *R*_pb_, where the variables are the identified composites and the biomass (data not shown). For the statistical analysis of the *Ganoderma* composites, we performed a McNemar Chi-squared test, where the variables were the composites in control and treatment of each species. This analysis was realized in the package ltm in the program R version 4.4.0 (https://www.R-project.org) [35, 36].

## 3. Results

### 3.1. Profile of VPW

The VPW phenolic profile showed hydroxybenzoic acids (16), hydroxycinnamic acids (4), flavonols (12), flavan-3-ols (5), stilbenes (13), flavones (6), flavonoids (4), and chalcones (2) (Table 1 and Table S1). Phenolic composites (PCs) were detected in all extracts. Due to its better performance, reverse-phase chromatography was used. Detection was standardized for most composites. The profile and concentration of PCs are distinguished among extracts. Ethanolic and aqueous extracts showed PC primary number and concentration. The main PCs in the ethanol extract were resveratrol dimer 4 (19.74 ± 0.49 mg/g), resveratrol tetramer 1 (16.39 ± 0.13 mg/g), naringenin (10.11 ± 0.65 mg/g), and naringenin-C-hexoside (10.41 ± 0.36 mg/g (Table S1). In the aqueous extract, the predominant composites were ellagic acid (4.45 ± 0.04 mg/g), quercitrin (3.99 ± 0.03 mg/g), quercetin dirhamnoside (3.88 ± 0.02 mg/g), and dihidroquercetin-3-O-rhamnoside (3.80 ± 0.00 mg/g). In the nonpolar extract's toluene and chloroform, the representative composites were caryatin (3.08 ± 0.00 and 2.98 ± 0.00 mg/g), naringenin (1.49 ± 0.03 and 3.09 ± 0.23 mg/g), and m-hydroxybenzoic acid (0.61 ± 0.02 and 1.06 ± 0.03 mg/g), respectively. Phenol diversity varied depending on the solvent. For example, aglycons and phenolic glycosides were more abundant in ethanolic and aqueous extracts.

In toluene and chloroform extracts, we identified 22 and 28 composites, respectively, 12 of them being shared, such as 2-propenoic acid, dodecanoic acid, and tetradecanoic acid (Table 2). The major composites in the toluene extract were hexadecanoic acid, octadecanoic acid, and trans-9-octadecenoic acid, while for the chloroform extract, hexadecanoic acid, tetradecanoic acid, and hexatriacontyl trifluoroacetate were used. It is the first approach to characterize the lipid and phenolic profile of VPW extracts (Figure S1).

**Figure 1:**
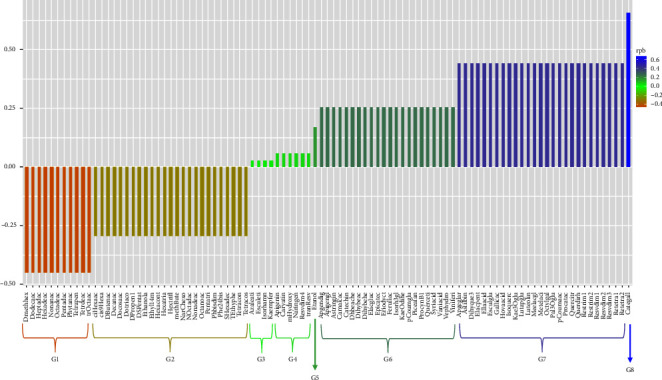
Correlation of vineyard pruning wastes extracts composites vs. biomass. G = group.

The correlation analysis between VPW extract composites and biomass (Figure 1) showed the highest value for catechin gallate (*R*_pb_ = 0.66), and group 7 has the second highest value (*R*_pb_ = 0.44) with 27 phenols mainly resveratrol tetramer 1, homovanillic acid, ellagic acid, and isoquercetin. Most are found in ethanol and aqueous extracts, except resveratrol trimer 1, only in ethanol.

### 3.2. Antiproliferative Activity

**Figure 2:**
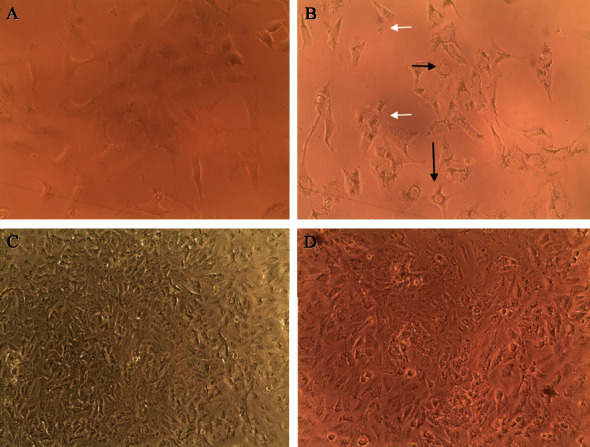
Morphological changes induced by *Ganoderma tuberculosum* extract of control at 15 d (GTC15) on A549 and ARPE-19 cell lines. (A) A549 cell line. (B) A549 + GTC15 24 h–200 µg/mL-20x. (C) ARPE-19 cell line. (D) ARPE-19 + GTC15 24 h–200 µg/mL-20x. Black arrows: formation of vacuoles; white arrows: cell debris.

The *G. lucidum* and *G. tuberculosum* mycelia ethanolic extracts showed different antiproliferative activity on cell lines (Table 3). *G. tuberculosum* ethanolic extracts showed a better antiproliferative effect on A549 at 15 days in both treatments; it could be accompanied by increased production of bioactive composites. Under these *Ganoderma* ethanolic extracts, no effect is observed in C-33 A and HeLa cancer cell lines. Morphological changes in A549 cells treated with the *G. tuberculosum* ethanolic extract were detected. Cell debris and vacuoles, which correspond to apoptotic and autophagy cell death, were observed (Figures 2(A) and 2(B)). The effect of *G. lucidum* ethanolic extracts was in the control at 15 days; it also affected C-33 A at 12 days with VPW and at 15 days with biomass control. *Ganoderma* species extracts (control and treatment) did not show an antiproliferative effect on the ARPE-19 noncancerous cell line nor morphological changes after treatment (Figure 2).

### 3.3. Identification of Ethanolic Extract Composites

**Figure 3:**
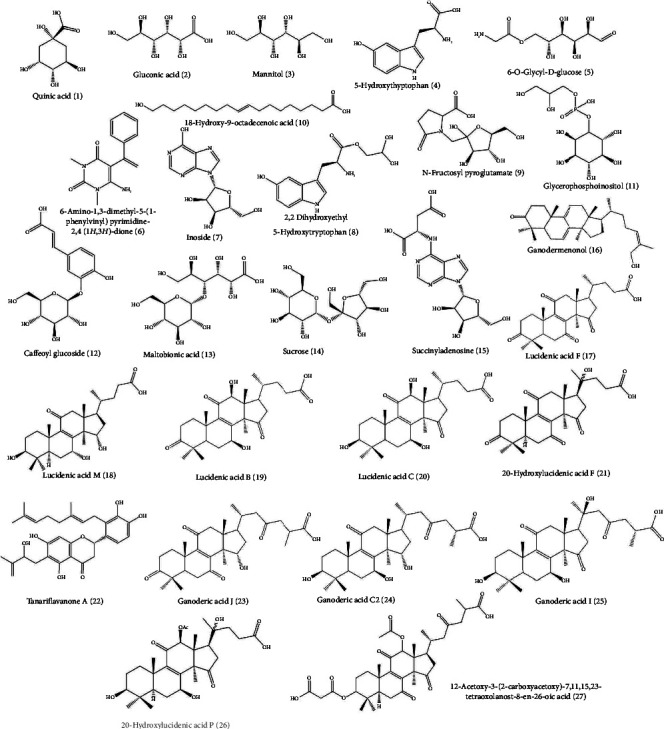
Composites identified in the extract of *Ganoderma lucidum* and *G. tuberculosum*.

**Figure 4:**
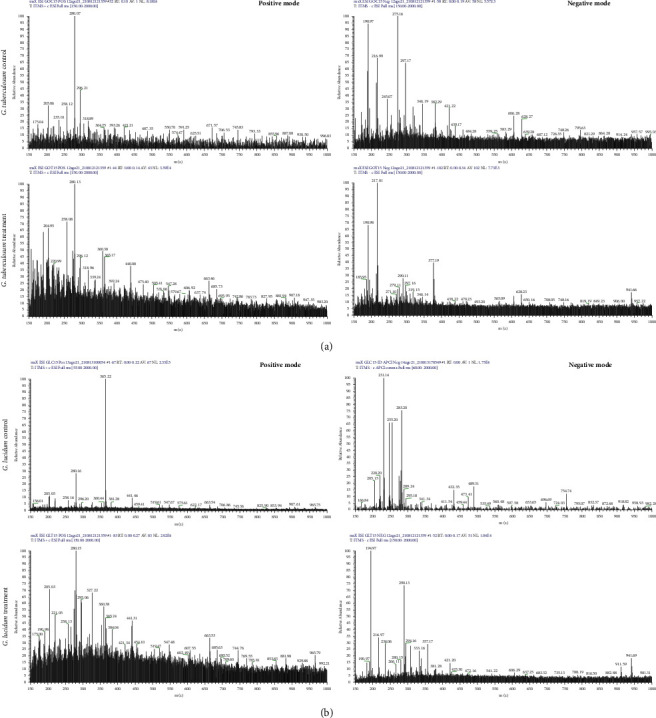
Chromatogram of *Ganoderma tuberculosum* and *G. lucidum ethanolic* extracts by ESI-MS in positive and negative mode, and APCI negative mode. (a) Chromatogram of ethanolic extracts of *G. tuberculosum* in control and treatment with VPW, (b) chromatogram of ethanolic extracts of *G. lucidum* in control and treatment with VPW.

Twenty-seven composites were identified, triterpenoids (11), organic acids (6), amino acids and derivates (5), sugars (3), fatty acid (1), and hydroxyflavanone (1) (Table 4, Figure 3). We found two classes of oxygenated lanostane-type triterpenoids, ganoderic, and lucidenic acids. Composites varied between species and treatments, but most were present in *G. tuberculosum* with VPW treatment. Ganodermenonol and 18-hydroxy-9-octadecenoic acid are present in *G. tuberculosum* control and treatment, meaning they are part of strain metabolism. Some composites are shared between species such as mannitol, quinic acid, 6-amino-1,3-dimethyl-5-(1-phenylvinyl)pyrimidine-2,4(1H,3H)-dione, and 2,2 dihydroxyethyl 5-hydroxytryptophan. Chromatogram differences between species and treatments are shown in Figure 4. However, the correlation analysis between identified compounds and species' treatments was insignificant.

### 3.4. Target-Prediction

Ganodermenonol, GA-I, and GA-C2 showed the capability to interact with the tumor necrosis factor (TNF)-*α* and nitric oxide synthase (NOS). Ganodermenonol (PubChem CID: 13934283) had a probability of 0.57 for TNF-*α* and 0.10 for DNA topoisomerase 2*α*. GA-I (PubChem CID: 73657195) had 0.90 and 0.14 for TNF-*α* and NOS, respectively. Lucidenic acid F (PubChem CID: 23247893) was 0.17 for NOS, 0.13 for 11*β*-hydroxysteroid dehydrogenase type 1, and 0.11 for TNF-*α*. GA-J (PubChem CID: 20055991) had 0.33 for TNF-*α*, 0.25 for squalene synthase, and 0.22 for NOS. GA-C2 (PubChem CID: 57396771) showed 0.85 for TNF-*α* and 0.19 for NOS.

### 3.5. Molecular Docking

**Figure 5:**
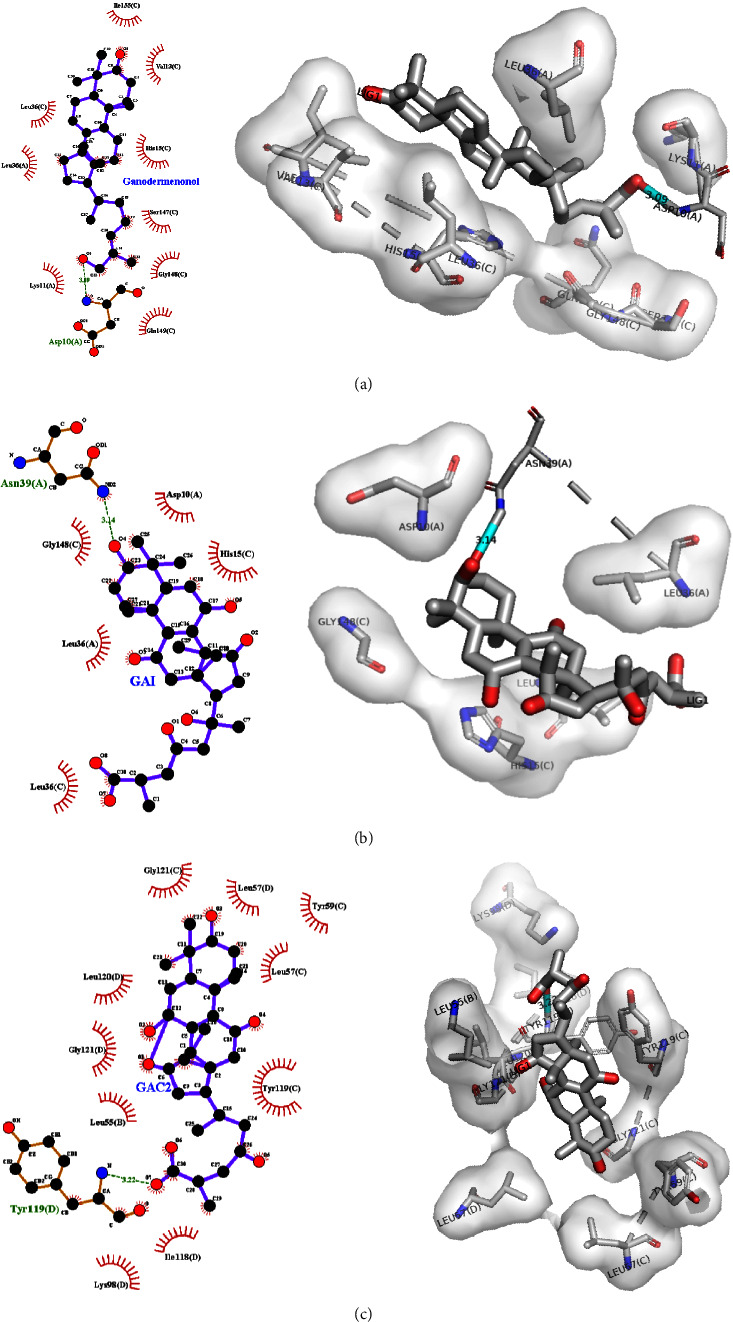
Molecular docking of ganodermenonol (a), ganoderic acid I (b), and ganoderic acid C2 (c) with TNF-*α* protein. Aqua: shows the H bond.

The capability of ganodermenonol, GA-I, and GA-C2 to interact with TNF-*α* was analyzed by molecular docking. The C23 carbonyl group of ganodermenonol makes a H bond (3.09 Å) with Asp10 of TNF-*α* chain A (ΔG of −7.68). In addition, ganodermenonol binds to chain C of TNF-*α* through six hydrophobic interactions with Ile155, Val13, His15, Ser147, Gly148, and Leu36 and interacts with chain A of TNF-*α* through two hydrophobic interactions with Lys11 and Leu36 (Figure 5(a)). The C23 carbonyl group of GA-I makes an H bond (3.14 Å) with Asn39 of TNF-*α* chain A (Δ*G* of −7.46). On the other hand, GA-I makes two hydrophobic interactions with Asp10 and Leu36 of TNF-*α* chain A and three hydrophobic interactions with His15, Leu36, and Gly148 of TNF-*α* chain C (Figure 5(b)). The C30 carbonyl group of GA-C2 interacts with Tyr119 of TNF-*α* chain D via the H bond (Δ*G* of −8.79, 3.22 Å). In addition, this compound makes four hydrophobic interactions with Gly121, Tyr59, Leu57, and Tyr119 of TNF-*α* chain C, five hydrophobic interactions with Leu120, Gly121, and Leu57, Lys98 of TNF-*α* chain D, and finally a hydrophobic interaction with Leu55 of TNF-*α* chain B (Figure 5(c)).

## 4. Discussion

Because different bioactive compounds are produced in the mycelium vs. fruiting body [55], strategies are required to enhance their production. To improve *Ganoderma* species' biomass and GAs production, we use VPW extracts as elicitors in liquid culture. In this study, the yield in VPW extracts was 0.79%, 0.142%, 1.74%, and 2.9% m/m toluene, chloroform, ethanol, and water, respectively, which are similar to Harris-Valle et al. [23]; the difference could be attributed to the storage time of VPW, which could cause the change in some chemical composites. The extract characterization showed the presence of PCs in the four extracts. The primary quality and quantity were for ethanol and aqueous extracts, but more studies are needed to know the non-PCs (N-PC) of toluene and chloroform extracts. *Vitis vinifera* VPW in different cultivars and industrial waste have been characterized to obtain by-products such as seed oil [56, 57]. Phenolic profiles of VPW extracts share some composites; for example, leaves and tendrils of ethanolic extracts [58] contain, like our study, gallic acid, catechin, epicatechin, isoquercitrin, quercitrin, and quercetin. The white grape waste extract showed PC, mainly catechin and gallic acid, lesser quercetin, 4-hydroxybenzoic acid, protocatechuic acid, and p-coumaric acid [57].

In nonpolar compounds of the VPW extract, a high percentage corresponds to fatty acids. Masuero et al. [59] analyzed the lipidomic of Ribolla Gialla grapes (*Vitis vinifera* L.) during maturation. They identified 412 composites, mainly glycerophospholipids and hydrozylates. Pardo et al. [56] mentioned that grape seed oil of five cultivars has different quantities of fatty acids such as myristic (14:0), palmitic (16:0), stearic (18:0), arachidic (20:0), oleic (18:1) linoleic (18:2), and linolenic (18:3). VPW lipid profile showed some fatty acids reported by Pardo et al. [56] such as tetradecanoic acid (myristic), hexadecanoic acid (palmitic), and octadecanoic acid (stearic). PC were found in VPW toluene and chloroform extracts (Table S1), such as phenols (Table 2). Miele et al. [60] studied the lipids of *Vitis vinifera* cv. Cabernet Sauvignon mainly contains linolenic acid, phospholipids, and linoleic acid in leaves, pericarps, and seeds, respectively.

VPW polar extracts improved the biomass production of *L. edodes* in the submerged culture but not with lipophilic compounds [23]. The lipid profile and biomass correlation were negative in *G. tuberculosum* and *G. lucidum* (*p*=0.4113). Evaluating the content of other lipophilic compounds such as esters, alkanes, and aldehydes in VPW toluene and chloroform extracts is necessary. Fatty acids are one of the essential composites in basidiomycetes due to the lignin degradation and lipid peroxidation, mainly in white-rot basidiomycetes such as *Ganoderma*; besides, peroxidizable plant lipids could also be used as a source [61]. Some VPW lipids could be assimilable molecules that improve the development of the *Ganoderma* liquid culture. Ganapathy et al. [17] mentioned that phenols could produce pores in the membrane due to their polarity, and gallic acid could produce H_2_O_2_, which increases ROS levels, disrupting fungal growth, but concentration could be relevant. The gallic acid content in ethanolic and aqueous extracts was 1.68 and 0.61 mg/g, respectively, which could be a target to be an elicitor in the mycelial culture, activating ROS and GAs pathway, as well as antioxidant enzymes. Many stimuli cause an increase in ROS, which could induce the GA synthesis [62].

The study presents a first approach to understand the elicitor composition of VPW extracts. VPW extracts enhance biomass production in liquid and solid cultures of *Ganoderma* spp. [22, 24]. We observed that 0.40 mg/g of catechin gallate in the ethanol extract could be most responsible for increasing the biomass in the liquid culture. Likewise, a synergistic effect of group 7 phenols (Figure 1) could occur to improve biomass production. Gallic acid is present in group 7, which has a positive correlation and could have the same performance reported by Ganapathy et al. [17] to stimulate ROS. Our results showed that ethanolic and aqueous extracts enhance biomass production. In future research, it will be necessary to study the metabolism of the interaction of each compound in the liquid culture of *Ganoderma*.

The antiproliferative capability of *G. lucidum* and *G. tuberculosum* extracts against cancer cell lines could not be optimal. Grever et al. [63] mentioned that an extract with an antiproliferative effect must have an IC_50_ < 30 *μg/mL* to be considered cytotoxic against cells. Therefore, whether the isolated composites have a better antiproliferative response is considered relevant. Hseu et al. [64] analyzed an ethanolic extract of *Ganoderma tsugae*, which, with 200–300 *μ*g/mL, showed an apoptotic and autophagic effect, the activity being dose-dependent. Ćilerdžić et al. [65] determined that extracts from three strains of *G. lucidum* have antiproliferative activity against the A549 cell line. The IC_50_ of these strains was 26.48, 167.01, and 95.87 *μ*g/mL for BEOFB431, BEOFB432, and BEOFB 434, respectively. These values varied from 100 to 170 *μ*g/mL, similar in this study with the same cell line (A549); this could be because both are ethanolic extracts, although coming from fruiting bodies vs. mycelium in the present study. Control and treated mycelium extracts showed the same antiproliferative capability against the A549 cell line. Li et al. [66] used similar concentrations of *Ganoderma leucocontextum* extracts (50–200 *μ*g/mL) and obtained an IC_50_ of 14–52 *μ*g/mL in antiproliferative activity vs. MDA-MB-231 cell line.

Martínez-Montemayor et al. [67] mentioned that an undefined *G. lucidum* extract, ReishiMax GLp, has antiproliferative activity, which may be associated with the synergistic effect of the unidentified compounds. Bioassay-guided isolation and chemical characterization of *Ganoderma* antiproliferative composites are crucial for discovering new composites. Li et al. [66] observed that ganoderiol F, an isolated compound in the fraction of the ethanolic extract of *G. leucocontextum*, has major effectivity on cancer cell lines (MDA-MB-231, SK-BR-3, MDA-MB-468, MCF-7, and 4T1) and a slight effect in the nontumorigenic epithelial MCF-10A cell line. Zhao and He [68] characterized a *G*. *lucidum* extract, identifying twelve composites in this extract by HPLC, EI-MS, and NRM. This extract shows a synergistic relation between GAs and tumor growth inhibition in Hepa1-6-bearing C57 BL/6 mice. Among these extracts, GA-C2 presents a synergistic score of 0.03 with GA-Y; some effects of extracts could be due to a synergy between the composites.

The vacuoles produced by *Ganoderma* extracts in the A549 cell line could be due to the autophagy mechanism, a survival strategy during cell destruction under stress conditions [69]; apoptosis and autophagy could occur. *G. tsugae* extracts against the K562 cell line demonstrate that autophagy is a dose-dependent survival mechanism activated through converting LC3-I to LC3-II [63]. Vacuole formation was present in the cancer cell line but not in ARPE-19; *Ganoderma* extracts could be possible selective extracts that do not present injury mechanisms in the nontumor cell line. The GAs interact with receptor tyrosine kinase (RTK) and modulate cancer pathways through the IR, IGFR-1, IGFR-2, VEGFR-1, VEFGR-2, and EGFR signaling networks. Healthy cells show no effects with ethanolic extracts. Healthy cells present DNase-dependent caspase (ICAD) inhibitors, while in apoptotic cells, DNase-dependent caspase (CAD) is active and begins chromatin fragmentation [70]. It is essential to test the compound in noncancerous cell lines to be sure that the apoptotic and cytotoxicity do not affect the healthy cell line. Compounds of *Ganoderma* extracts affect cancer cell lines due to their interaction with membrane receptors, leading to apoptosis through different signals.

The composites identified from mycelial ethanolic extracts of both species differ from VPW extract composites. The antiproliferative effect seems limited to *Ganoderma* bioactivity. The composites shared between *G. tuberculosum* and *G. lucidum* with and without VPW extracts generally correspond to primary metabolisms such as mannitol, sucrose, amino acids, and organic acids [71, 72]. Different compounds were also found between the species evaluated, confirming the variability of bioactive molecules in *Ganoderma* species. The evaluated treatment of VPW extracts enhanced the biomass production in the liquid culture of *Ganoderma* spp.; however, the production of bioactive composites was not increased. Treatments should be changed to achieve possible enhancement in GAs production. Keypour et al. [73] mentioned differences in the chemical composites due to geographical distributions, growth conditions, and substrates. They identified four GAs (GA-H, GA-T, GA-Me, and GA-C2) in two *G. lucidum* strains from Iran and China. It is known that not all composites have antiproliferative activity.

Some studies demonstrated the bioactivity of some composites that we identified. GA-C2 exhibits antitumor, cytotoxic, antihistamine, antiaging, and antidiabetic activity; it is also undergoing preclinical treatment for aldose reductase inhibition at Dalian Medical University and Peking University. GA-C2 has a double bond in C-22 and C-20 and a -OH substituent at C-3, which are necessary for maintaining *α*-glucosidase inhibition [7]. Huang et al. [74] mentioned that ganodermenonol demonstrated cytotoxic activity against HeLa and HeLa/VCR cell lines with IC_50_ 44.70 ± 2.32 and 41.33 ± 2.15, respectively. Our extracts did not show antiproliferative activity in HeLa cell lines; this could be because they are from a different biological source and concentration; we used the ethanolic extract of mycelium vs. fruiting bodies in the previous research [75]. The pharmacological properties of some GAs determined in this study are still unknown.

Target prediction analysis demonstrates some targets for these GAs, such as TNF-*α*, NOS, 11*β*-hydroxysteroid dehydrogenase I, or DNA polymerase I. Some results show targets of the mevalonate pathway, which could indicate negative feedback during GA synthesis. HMGR exhibits this response with the farnesyl and ergosterol in yeast [76, 77]. Molecular docking also showed interactions between TNF-*α* and GAs with Tyr119, Asp10, and Asn39 residues. The GAs skeleton has a role in signaling cascades in some mitogenic pathways. In GA A, C2, R, H, T, E, B, S, F, and DM, the H-bonding residues of TNF-*α* are Tyr151, Leu120, and Gln149. GA-A efficiently inhibits cell proliferation through intracellular ROS in breast cancer cell lines [78].

## 5. Conclusions

This first characterization of VPW extracts showed catechin gallate improves biomass in the *Ganoderma* liquid culture. Still, it is necessary to isolate the composite to demonstrate an individual or synergistic effect. The *G. tuberculosum* mycelial ethanolic extracts, a native strain from cactus of the Sonoran Desert, contain at least 17 composites such as ganoderic and lucidenic acids, sugars, fatty acids, and organic acids. Furthermore, these extracts showed antiproliferative activity and induced vacuole formation in the A549 cell line without damaging healthy cell lines; this selectivity could have pharmaceutical relevance. Molecular docking shows interaction with hydrogen bonds and nonligand residues with hydrophobic contacts for GA I, C2, and ganodermenonol vs. TNF-*α*, indicating a possible effect antiproliferative and/or anti-inflammatory.

## Figures and Tables

**Table 1:** Major PCs in ethanol and aqueous extracts of VPW.

**Composites**	**R.T. (min)**	**Formula**	**Precursor**[**M** − **H**]^−^	**Experimental mass**	**Theoretical mass**	**Score**	**Difference (ppm)**	**Fragments (** **M** **MS** ^2^ **)**	**Ethanol (mg/g)**	**Aqueous (mg/g)**
Hydroxybenzoic acids										
Gallic acid	0.41	C_7_ H_6_ O_5_	169.0149	170.0221	170.0215	95.93	3.51	169.0141; 125.0204	1.38 ± 0.04	0.61 ± 0.03
*m*-Hydroxybenzoic acid	1.08	C_7_ H_6_ O_3_	137.0240	138.0312	138.0317	79.93	−3.72	137.0243	1.59 ± 0.05	1.85 ± 0.00
Ellagic acid	3.65	C_14_ H_6_ O_8_	300.9989	302.0062	302.0063	97.14	−0.23	300.9989; 229.0135; 129.0360	4.11 ± 0.09	4.45 ± 0.04
Ellagic acid pentoside	3.41	C_19_ H_14_ O_12_	433.0407	434.048	434.0485	98.21	−1.33	433.0407; 300.9972; 299.9918	2.37 ± 0.04	3.65 ± 0.08
Methyl ellagic acid	4.52	C_15_ H_8_ O_8_	315.0146	316.0219	316.0219	85.14	−0.12	315.0146	0.79 ± 0.02	2.11 ± 0.02
Methyl ellagic acid glucoside	3.81	C_21_ H_18_ O_13_	477.0676	478.0753	478.0747	96.08	−1.28	477.0635; 301.0338	1.23 ± 0.01	2.11 ± 0.02
Protocatechuic acid	0.68	C_7_ H_6_ O_4_	153.0195	154.0268	154.0266	92.00	0.98	153.0167; 109.0270	2.40 ± 0.06	2.06 ± 0.00
Homovanillic acid	4.42	C_9_ H_10_ O_4_	181.0499	182.0572	182.0579	84.51	−3.80	181.0489; 135.0443	4.36 ± 0.13	2.75 ± 0.12
Stilbenes										
*trans*-Resveratrol	5.47	C_14_ H_12_ O_3_	227.0712	228.0785	228.0786	99.73	−0.63	227.0711; 207.0664	1.51 ± 0.01	1.45 ± 0.00
Resveratrol dimer 1	4.52	C_28_ H_22_ O_6_	453.1359	454.1422	454.1416	98.83	1.22	453.1335; 359.0923; 315.0976	2.17 ± 0.02	1.60 ± 0.01
Resveratrol dimer 2	5.03	C_28_ H_22_ O_6_	453.1345	454.1418	454.1416	98.97	0.35	453.1345; 359.0920; 289.0867	1.60 ± 0.03	1.47 ± 0.03
Resveratrol dimer 3	5.33	C_28_ H_22_ O_6_	453.1355	454.1426	454.1416	97.30	−2.12	453.1346; 359.1328	1.58 ± 0.05	1.42 ± 0.00
Resveratrol dimer 4	5.87	C_28_ H_22_ O_6_	453.1349	454.1422	454.1416	97.23	−1.36	453.1346; 427.1541	19.74 ± 0.49	2.40 ± 0.03
Resveratrol trimer 2	5.97	C_42_ H_32_ O_9_	679.1974	680.2045	680.2046	98.98	−0.13	679.1971; 637.840; 585.1543	2.88 ± 0.24	1.43 ± 0.00
Resveratrol tetramer 1	5.63	C_56_ H_42_ O_12_	905.2615	906.2688	906.2676	96.04	−1.35	905.3587; 811.2158	16.39 ± 0.13	1.41 ± 0.00
Resveratrol tetramer 2	6.41	C_56_ H_42_ O_12_	905.2605	906.2677	906.2676	99.46	−0.08	905.2601; 811.2185	1.47 ± 0.01	1.74 ± 0.01
Pallidol-3-O-glucoside	5.13	C_34_ H_32_ O_11_	615.1877	616.1947	616.1945	96.38	−0.38	615.1900; 453.1343; 359.0865	1.59 ± 0.02	1.51 ± 0.00
Flavonols										
Kaempferol-3-O-glucoside	3.31	C_21_ H_20_ O_11_	447.0920	448.0993	448.1006	78.14	−2.85	447.0924; 285.0386; 285.0386	3.31 ± 0.02	3.47 ± 0.02
Quercitrin	3.17	C_21_ H_20_ O_11_	447.0934	448.1011	448.1006	86.42	1.09	447.0945; 357.0609; 327.0500	3.58 ± 0.03	3.99 ± 0.03
Quercetin dirhamnoside	4.05	C_27_ H_30_ O_15_	593.1528	594.1590	594.1585	75.58	0.91	593.1512	3.33 ± 0.00	3.88 ± 0.02
Dihydroquercetin-3-O-rhamnoside	3.78	C_21_ H_22_ O_11_	449.1091	450.1172	450.1162	75.73	2.27	449.2028; 269.1399; 151.0034	3.79 ± 0.00	3.80 ± 0.00
Caryatin	5.87	C_17_ H_14_ O_7_	329.0667	330.0741	330.0740	95.60	0.29	329.0667	3.34 ± 0.01	< L.O.Q. ∗
Isoquercetin	3.85	C_21_ H_20_ O_12_	463.0881	464.0955	464.0955	97.52	0.07	463.857; 301.0326; 300.027	3.88 ± 0.01	3.87 ± 0.01
Flavanones										
Astilbin	3.92	C_21_ H_22_ O_11_	449.1088	450.1169	450.1162	82.61	1.55	449.1195; 269.0433; 179.0356	0.69 ± 0.00	0.73 ± 0.00
Naringenin	5.84	C_15_ H_12_ O_5_	271.0615	272.0685	272.0685	92.66	−0.03	271.0612	10.11 ± 0.65	1.81 ± 0.03
Flavones										
Apigenin	5.83	C_15_ H_10_ O_5_	269.0453	270.0524	270.0528	77.63	−1.65	269.0445	3.01 ± 0.00	< L.O.Q. ∗
Apigenin-C-glucoside-8-C-arabinoside	2.97	C_26_ H_28_ O_14_	563.1405	564.1467	564.1479	86.71	−2.11	563.1456; 472.1089; 353.0610	3.04 ± 0.01	3.41 ± 0.01

^∗^L.O.Q. = < limit of quantification.

**Table 2:** Lipid profile of nonpolar extracts of VPW.

**Peak**	**Composites**	**Ret. time**	**Area (%)**	**Height (%)**	**Similarity**	**Formula**	**Molecular weight (g/mol)**
*Toluene*							
1	Octanoic acid	18.416	0.68045563	0.29109342	78	C_8_H_16_O_2_	144.2114^1^
2	Phenol, 2,4-*bis*(1,1-dimethylethyl)/2,4-di-tert-butylphenol	19.313	0.14066829	0.1310748	77	C_14_H_22_O	206.3239^1^
3	Nonanoic acid	21.521	0.38602442	0.46688874	89	C_9_H_18_O_2_	158.238^1^
4	3-Ethylphenol	22.478	0.18335538	0.17619421	76	C_8_H_10_O	122.1644^1^
5	Decanoic acid	24.476	0.18497287	0.2333288	82	C_10_H_20_O_2_	172.2646^1^
6	2-Propenoic acid	24.783	0.15590222	0.22526524	81	C_3_H_4_O_2_	72.0627^1^
7	Methyl but-2-enoate/2-butenoic acid	29.204	1.40474179	1.92164754	78	C_5_H_8_O_2_	100.1158^1^
8	Dodecanoic acid	29.995	0.35723322	0.48865625	89	C_12_H_24_O_2_	200.3178^1^
9	Tetradecanoic acid	35.032	5.87646694	6.46627482	92	C_14_H_28_O_2_	228.3709^1^
10	Pentadecanoic acid	37.393	0.96547345	1.36245843	93	C_15_H_30_O_2_	242.3975^1^
11	*cis*-9-Hexadecenoic acid	39.239	1.7322307	1.3652954	88	C_16_H_30_O_2_	254.4082^1^
12	Hexadecanoic acid	39.691	45.0205329	48.117537	90	C_16_H_32_O_2_	256.4241^1^
13	Heptadecanoic acid	41.837	2.1150458	2.48331574	86	C_17_H_34_O_2_	270.4507^1^
14	2-Methylhexacosane	42.138	0.37662957	0.49066562	86	C_27_H_56_	380.7335^1^
15	*trans*-9-Octadecenoic acid	43.466	12.3390525	10.1399332	91	C_18_H_34_O_2_	282.4614^1^
16	Octadecanoic acid	44.059	18.4719699	18.602847	93	C_18_H_36_O_2_	284.4772^1^
18	Nonadecanoic acid	46.664	0.24082373	0.25933127	77	C_19_H_38_O_2_	298.5038^1^
19	Tetrapentacontane	47.019	0.87458882	0.70826992	88	C_54_H_110_	759.4512^1^
20	Phytanic acid	49.89	4.85117981	3.7369366	87	C_20_H_40_O_2_	312.5^2^
21	Pentatriacontane	50.31	2.05264799	1.51235566	90	C_35_H_72_	492.9462^1^
22	Tetracontane-1,40-diol	56.221	1.59000408	0.8206304	88	C_40_H_82_O_2_	595.1^2^

*Chloroform*							
1	2-Propen-1-ol	12.421	0.65785094	0.50476533	70	C_3_H_6_O	58.08^2^
2	Ethanolamine	17.586	1.15330143	1.10567683	71	C_2_H_7_NO	61.08^2^
3	Phenol, 3,5-*bis*(1,1-dimethylethyl)	19.3	0.29515818	0.45371619	77	C_14_H_22_O	206.3239^1^
4	Nonanoic acid	21.515	0.3034953	0.50004382	84	C_9_H_18_O_2_	158.238^1^
5	2-Butenoic acid	22.326	0.44576813	0.84743414	86	C_4_H_6_O_2_	86.0892^1^
6	3-Methyl but-2-enoate	29.192	2.59733748	5.44644432	78	C_5_H_7_O_2_^−^	99.11^2^
7	Dodecanoic acid	29.981	0.28378861	0.51941537	86	C_12_H_24_O_2_	200.3178^1^
8	Heptadecanoic acid	32.852	0.72754323	1.5478404	85	C_17_H_34_O_2_	270.4507^1^
9	Tetradecanoic acid	35.021	22.1790421	7.891303	78	C_14_H_28_O_2_	228.3709^1^
10	7-Hexadecenal	36.756	0.77343821	1.16608526	83	C_16_H_30_O	238.41^2^
11	9-Octadecenoic acid	37.026	0.71853177	1.14847171	79	C_18_H_34_O_2_	282.4614^1^
12	Pentadecanoic acid	37.381	0.38779319	0.72829158	83	C_15_H_30_O_2_	242.3975^1^
13	Docosanoic acid	37.63	0.24422573	0.51317864	78	C_22_H_44_O_2_	340.5836^1^
14	2-Methylhexacosane	37.756	0.13012606	0.33802266	85	C_27_H_56_	380.7335^1^
15	*cis*-9-Hexadecenoic acid	39.229	1.13552978	1.35978401	84	C_16_H_30_O_2_	254.4082^1^
16	Hexadecanoic acid	39.655	17.9771098	33.1669981	93	C_16_H_32_O_2_	256.4241^1^
17	Heptadecanoic acid	41.828	0.8456943	1.35998492	81	C_17_H_34_O_2_	270.4507^1^
18	Tetrapentacontane	42.129	0.82384475	1.48668691	88	C_54_H_110_	759.4512^1^
19	*trans*-9-Octadecenoic acid	43.456	8.26244838	9.7355097	90	C_18_H_34_O_2_	282.4614^1^
20	Octadecanoic acid	44.027	5.97427243	9.97883445	93	C_18_H_36_O_2_	284.4772^1^
21	Hexacontane	44.346	2.42277647	3.13957235	80	C_60_H_122_	843.6107^1^
22	17-Pentatriacontene	45.587	1.28601144	1.13932171	79	C_35_H_70_	490.9303^1^
23	Ethyl 14-methyl-hexadecanoate	46.877	0.64706454	1.01004968	73	C_19_H_38_O_2_	298.5^2^
24	Dotriacontane	47.001	1.9378505	2.6136691	87	C_32_H_66_	450.8664^1^
25	Phytanic acid	49.872	1.85235341	2.31825715	84	C_20_H_40_O_2_	312.5^2^
26	Hexatriacontane	50.282	2.13917871	2.34540578	87	C_36_H_74_	506.9728^1^
27	Tetracosanoic acid	53.576	9.58264275	3.67146119	87	C_24_H_48_O_2_	368.6367^1^
28	Hexatriacontyl trifluoroacetate	54.614	14.2158224	3.9637757	77	C_38_H_73_F_3_O_2_	618.9802^1^

^1^NIST.

^2^PubChem databases used to confirm formula and molecular weight.

**Table 3:** Antiproliferative activity of *Ganoderma* species ethanolic extracts versus cell lines.

**Extracts**	**Cell lines** **I** **C**_50_ **(*μ*g/mL)**			
	**A549**	**C-33 A**	**HeLa**	**ARPE-19**
GLC12	> 200	> 200	> 200	> 200
GLT12	> 200	175.2 ± 3.9	> 200	> 200
GLC15	145.2 ± 4.8	191.2 ± 16.7	> 200	> 200
GLT15	> 200	> 200	> 200	> 200
GTC12	176.3 ± 13	> 200	> 200	> 200
GTT12	> 200	> 200	> 200	> 200
GTC15	138.1 ± 11.7	> 200	> 200	> 200
GTT15	143.2 ± 12.8	> 200	> 200	> 200

*Note:* IC_50_ results are expressed in *μ*g/mL and represent the average of three independent experiments with standard deviation (±SD).

Abbreviations: GLC, *G. lucidum* control; GLT, *G. lucidum* treatment; GTC, *G. tuberculosum* control; GTT, *G. tuberculosum* treatment; 12 and 15, mycelium culture days.

**Table 4:** The *G. tuberculosum* and *G. lucidum* ethanolic extract compounds.

**No**	**Composite**	**m/z**	**Adduct**	**MSn**	**GLC**	**GLT**	**GTC**	**GTT**	**Reference**
1	Quinic acid	191	(M − H)^−^	173, 127, 111, and 85	•	•	•	•	[37]
2	Gluconic acid	195	(M − H)^−^	177, 159, 129, 99, and 87	•	•			[38]
3	Mannitol	205/217	(M + Na)^+^/(M + Cl)^−^	188, 187, 173, 159, 146, 145, and 131	•	•	•	•	[39, 40]
4	5-Hydroxytryptophan	221	(M + H)^+^	203, 177, 147, and 133	•	•			[41, 42]
5	6-O-Glycyl-D-glucose	236	(M − H)^−^	218, 208, 164, 115, and 74		•			[43]
6	6-Amino-1,3-dimethyl-5-(1-phenylvinyl)pyrimidine-2,4(1H,3H)-dione	258	(M + H)^+^	227, 199, 181, 171, 155, 140, 141, and 104	•		•	•	[44]
7	Inoside	266	(M − H)^−^	248, 194, 176, 134, and 104		•			[43]
8	2,2 dihydroxyethyl 5-hydroxytryptophan	280	(M + H)^+^	221, 104 ⟶ 177, and 147	•	•	•	•	[41, 42]
9	N-Fructosyl pyroglutamate	290	(M − H)^−^	272, 254, 230, 214, 200, 170, 154, and 128		•		•	[45]
10	18-Hydroxy-9-octadecenoic acid	297	(M − H)^−^	279 and 155			•	•	[46]
11	Glycerophosphoinositol	333	(M − H)^−^	241, 171, 153, and 97		•			[47]
12	Caffeoyl glucoside	341	(M − H)^−^	179, 161, 149, 141, and 131	•			•	[37]
13	Maltobionic acid	357	(M − H)^−^	339, 321, 313, 179, and 161		•			[43]
14	Sucrose	365	(M + Na)^+^	203 and 185	•	•	•	•	[39, 48]
15	Succinyladenosine	382	(M − H)^−^	266, 206, and 134			•		[43]
16	Ganodermenonol	439	(M + H)^+^	421, 406, 309, and 269			•	•	[49]
17	Lucidenic acid F	456	(M − H)^−^	437, 395, 383, 380, and 377		•			[50]
18	Lucidenic acid M	461	(M − H)^−^	443, 417, and 319			•		[46]
19	Lucidenic acid B	473	(M − H)^−^	443 and 330	•				[43]
20	Lucidenic acid C	475	(M − H)^−^	457, 445, 439, 427, and 413				•	[46]
21	20-Hydroxylucidenic acid F	495	(M + Na)^+^	477, 463, 451, and 349				•	[51]
22	Tanariflavanone A	507	(M − H)^−^	489 and 403			•		[46]
23	GA J	515	(M + H)^+^	497, 479, and 367	•				[52]
24	GA C2	519	(M + H)^+^	501 and 483				•	[53]
25	GA I	*533*	(M + H)^+^	515 and 497				•	[54]
26	20-Hydroxylucidenic acid P	535	(M + H)^+^	517, 503, 491, 473, and 307				•	[51]
27	12-Acetoxy-3-(2-carboxyacetoxy)-7,11,15,23-tetraoxolanost-8-en-26-oic acid	657	(M − H)^−^	639, 595, 553, and 467		•			[46]

Abbreviations: GLC, *G. lucidum* control extract; GLT, *G. lucidum* treatment extract; GTC, *G. tuberculosum* control extract; GTT, *G. tuberculosum* treatment extract.

## Data Availability

The data used to support the findings of this study are included within the article.

## Supporting Information

Supporting Information Table Supplementary 1: the minority of phenolic compounds in vineyard pruning waste extracts.

Supporting Information Table Supplementary 2: linearity information of calibration curves.

Supporting Information Figure Supplementary 1: chromatogram of the lipid profile of the nonpolar extracts of vineyard pruning. (a) Chromatogram of the toluene extract of vineyard pruning extracts by GC-MS. (b) Chromatogram of the chloroform extract of vineyard pruning extracts by GC-MS.
