# Supplementary material for: Ganoderma tuberculosum Liquid Culture With Vineyard Pruning Extracts for Bioactive Composite Production With Antiproliferative Activity
Source: Adv Pharmacol Pharm Sci. 2024 Oct 24;2024:5245451. doi: 10.1155/2024/5245451 (PMC11527534; doi:10.1155/2024/5245451)
Supplement: Supporting Information — Table Supplementary 1: the minority of phenolic compounds in vineyard pruning waste extracts. [file 5245451.f1.docx]

## Supplementary Material

***Ganoderma oerstedii* liquid culture with vineyard pruning extracts for bioactive composite production with antiproliferative activity**

Lucia T. Angulo-Sanchez^1^, María C. Cruz-Félix^1^, Max Vidal-Gutiérrez^2^, Heriberto Torres-Moreno^3^, Óscar Adrián Muñoz-Bernal^4^, Emilio Álvarez-Parrilla^4^, Ramón Enrique Robles-Zepeda^5^, Osiris Álvarez-Bajo^6^, Aldo Gutiérrez^1^ and Martín Esqueda^1,*^

^1^ Centro de Investigación en Alimentación y Desarrollo, A.C. Carretera Gustavo Enrique Astiazarán Rosas 46, La Victoria, CP. 83304, Hermosillo, Sonora, México.

^2^ Universidad de Sonora. Campus Navojoa, Departamento de Ciencias Químico, Biológicas y Agrope-cuarias, Lázaro Cárdenas del Río 100, Francisco Villa, CP. 85880, Navojoa, Sonora, México.

^3^ Universidad de Sonora. Campus Caborca, Departamento de Ciencias Químico, Biológicas y Agrope-cuarias, Avenida K SN, Eleazar Ortiz, CP. 83600, H. Caborca, Sonora, México.

^4^ Universidad Autónoma de Ciudad Juárez. Instituto de Ciencias Biomédicas. Av. Benjamín Franklin 4650, Condominio La Plata, CP. 32310, Ciudad Juárez, Chihuahua, México.

^5^ Universidad de Sonora. Campus Hermosillo, Departamento de Ciencias Químico Biológicas, Blvd. Luis Donaldo Colosio y Rosales s/n, Centro, CP. 83000, Hermosillo, Sonora, México.

^6^ Consejo Nacional de Ciencia y Tecnología-Universidad de Sonora. Blvd. Luis Encinas y Rosales s/n, CP. 83000, Hermosillo, Sonora, México.

*Corresponding author: esqueda@ciad.mx, tel.: +52 (662) 289-2400

Table Supplementary 1. The minority of phenolic composites in vineyard pruning waste extracts.

| Composites | R.T. (min) | Formula | Precursor  [M-H]^-^ | Experimental mass | Theorical Mass | Difference (ppm) | Fragments (MS^2^) | Toluene  (mg /g) | Chloroform (mg /g) | Ethanol  (mg /g) | Aqueous  (mg /g) |
| --- | --- | --- | --- | --- | --- | --- | --- | --- | --- | --- | --- |
| Hydroxybenzoic acids |  |  |  |  |  |  |  |  |  |  |  |
| *m*-Hydroxybenzoic acid | 1.08 | C_7_ H_6_ O_3_ | 137.0240 | 138.0312 | 138.0317 | -3.72 | **137.0243**; | 0.61 ± 0.02 | 1.06 ± 0.03 | 1.59 ± 0.05 | 1.85 ± 0.00 |
| 2-O-*p*-hydroxybenzoyl-6-O-galloyl-glucoside | 4.96 | C_20_ H_20_ O_12_ | 451.0901 | 452.0973 | 452.0955 | -3.98 | 451.0892; **359.0917**; 331.0667 | N.D. | N.D. | N.D. | 0.83 ± 0.08 |
| Dihydroxybenzoyl hexose | 5.23 | C_20_ H_20_ O_11_ | 435.0935 | 436.1008 | 436.1006 | -0.50 | **435.0902**; | N.D. | N.D. | N.D. | <L.O.Q. |
| Dihydroxybenzoic acid | 1.26 | C_7_ H_6_ O_4_ | 153.0188 | 154.0260 | 154.0266 | -3.60 | **153.0199**; | N.D. | N.D. | <L.O.Q. | N.D. |
| Dihydroxybenzoic acid hexose | 0.36 | C_13_ H_16_ O_9_ | 435.0935 | 436.1008 | 436.1006 | -0.50 | **435.0902**; | N.D. | N.D. | <L.O.Q. | N.D. |
| Ellagic acid glucoside | 2.26 | C_20_ H_16_ O_13_ | 463.0513 | 464.0587 | 464.0591 | -0.75 | **463.0503**; 333.0603 | N.D. | N.D. | N.D. | 1.24 ± 0.05 |
| Syringic acid | 2.94 | C_9_ H_10_ O_5_ | 197.0453 | 198.0527 | 198.0528 | -0.29 | **197.0457**; | N.D. | N.D. | N.D. | 0.51 ± 0.04 |
| Vanillic acid | 4.79 | C_8_ H_8_ O_4_ | 329.0877 | 330.0947 | 330.0951 | -1.18 | **329.0886**; 121.0297 | N.D. | N.D. | 0.35 ± 0.02 | N.D. |
| Hydroxycinnamic acids |  |  |  |  |  |  |  |  |  |  |  |
| Caffeic acid | 0.78 | C_9_ H_8_ O_4_ | 179.0349 | 180.0424 | 180.0423 | 0.68 | 179.0348; **135.0448**; 119.0363 | N.D. | N.D. | N.D. | N.D. |
| Caffeoylmalic acid | 1.22 | C_13_ H_12_ O_8_ | 295.0458 | 296.0530 | 296.0532 | -0.78 | **295.0471**; 247.0242 | N.D. | N.D. | N.D. | <L.O.Q. |
| Ferulic acid | 4.69 | C_10_ H_10_ O_4_ | 193.0502 | 194.0575 | 194.0579 | -2.24 | 193.0502; 165.0171; **134.0365** | N.D. | N.D. | <L.O.Q. | N.D. |
| *p*-Coumaric acid | 1.26 | C_9_ H_8_ O_3_ | 163.0396 | 164.0468 | 164.0473 | -3.08 | **163.0451**; 119.0542 | N.D. | N.D. | <L.O.Q. | 0.24 ± 0.01 |
| *p*-Coumaroyl-glucoside | 1.10 | C_15_ H_18_ O_8_ | 325.0938 | 326.1008 | 326.1002 | 1.78 | 325.1859; 163.0409; **119.0495** | N.D. | N.D. | <L.O.Q. | N.D. |
| Stilbenes |  |  |  |  |  |  |  |  |  |  |  |
| *trans*-Resveratrol | 5.47 | C_14_ H_12_ O_3_ | 227.0712 | 228.0785 | 228.0786 | -0.63 | 227.0711; **207.0664** | <L.O.Q. | <L.O.Q. | 1.51 ± 0.01 | 1.45 ± 0.00 |
| Resveratrol dimer 4 | 5.87 | C_28_ H_22_ O_6_ | 453.1349 | 454.1422 | 454.1416 | -1.36 | **453.1346**; 427.1541 | <L.O.Q. | 3.37 ± 0.08 | 19.74 ± 0.49 | 2.40 ± 0.03 |
| Astringin | 3.78 | C_20_ H_22_ O_9_ | 405.1202 | 406.1274 | 406.1264 | 2.37 | **405.1732**; | N.D. | N.D. | <L.O.Q. | N.D. |
| *alpha-*Viniferin | 6.13 | C_42_ H_30_ O_9_ | 677.1811 | 678.1891 | 678.1890 | -0.12 | **677.1868**; 571.1319; 465.0836 | N.D. | N.D. | N.D. | 1.42 ± 0.00 |
| Flavan-3-ols |  |  |  |  |  |  |  |  |  |  |  |
| Catechin | 1.09 | C_15_ H_14_ O_6_ | 289.0719 | 290.0792 | 290.0790 | -0.56 | 289.0720; 247.0299; **137.0251** | N.D. | N.D. | 1.40 ± 0.05 | N.D. |
| Epicatechin | 1.89 | C_15_ H_14_ O_6_ | 289.0720 | 290.0792 | 290.0790 | 0.19 | 289.0714; **247.0239**; 205.0506 | N.D. | N.D. | <L.O.Q. | N.D. |
| Catechin gallate | 3.81 | C_22_ H_18_ O_10_ | 441.0838 | 442.0914 | 442.0900 | 3.19 | 441.1984; 347.1004; **289.0746** | N.D. | N.D. | 0.40 ± 0.00 | N.D. |
| Procyanidin B1 | 0.95 | C_30_ H_26_ O_12_ | 577.1345 | 578.1411 | 578.1424 | -2.23 | 577.1243; **407.0784**; 289.0686 | N.D. | N.D. | <L.O.Q. | N.D. |
| B-Type procyanidin trimer | 3.68 | C_45_ H_38_ O_18_ | 865.1987 | 866.2051 | 866.2058 | -0.78 | **865.1969**; 289.0714; | N.D. | N.D. | N.D. | N.D. |
| Flavonols |  |  |  |  |  |  |  |  |  |  |  |
| Caryatin | 5.87 | C_17_ H_14_ O_7_ | 329.0667 | 330.0741 | 330.0740 | 0.29 | **329.0667** | 3.08 ± 0.00 | 2.98 ± 0.00 | 3.34 ± 0.01 | <L.O.Q. |
| Trifolin | 4.22 | C_21_ H_20_ O_11_ | 447.0933 | 448.1007 | 448.1006 | 0.27 | **447.0929**; 415.1955 | N.D. | N.D. | N.D. | N.D. |
| Kaempferol-O-dihexoside | 3.71 | C_27_ H_30_ O_16_ | 609.1461 | 610.1534 | 610.1534 | 0.06 | **609.1453**; 301.0354 | N.D. | N.D. | 3.08 ± 0.00 | N.D. |
| Quercetin | 5.33 | C_15_ H_10_ O_7_ | 447.0934 | 448.1011 | 448.1006 | 1.09 | 447.0945; 357.0609; **327.0500** | N.D. | N.D. | 3.98 ± 0.01 | N.D. |
| Azaleatin | 5.97 | C_16_ H_12_ O_7_ | 315.0511 | 316.0584 | 316.0583 | 0.36 | **315.050** | <L.O.Q. | N.D. | <L.O.Q. | N.D. |
| Isorhamnetin | 6.00 | C_16_ H_12_ O_7_ | 315.0512 | 316.0586 | 316.0583 | 1.03 | **315.0510** | N.D. | <L.O.Q. | 3.36 ± 0.01 | N.D. |
| Isorhamnetin-3-O-glucoside | 4.29 | C_22_ H_22_ O_12_ | 477.1037 | 478.1119 | 478.1111 | 1.70 | **477.1013**; 314.0425; 243.0377 | N.D. | N.D. | 3.40 ± 0.01 | N.D. |
| Flavanones |  |  |  |  |  |  |  |  |  |  |  |
| Eriodictyol | 4.45 | C_15_ H_12_ O_6_ | 287.0565 | 288.0637 | 288.0634 | 0.94 | 287.0565; 151.0017; **135.0448** | N.D. | N.D. | 0.68 ± 0.01 | N.D. |
| Hesperetin | 6.00 | C_16_ H_14_ O_6_ | 301.0717 | 302.0786 | 302.0790 | -1.23 | **301.0718** | N.D. | N.D. | N.D. | N.D. |
| Naringenin-C-hexoside | 3.95 | C_21_ H_22_ O_10_ | 433.1139 | 434.1211 | 434.1213 | -0.45 | 433.1100; **271.0617**; 227.0718 | N.D. | N.D. | 10.41 ± 0.36 | N.D. |
| Naringenin | 5.84 | C_15_ H_12_ O_5_ | 271.0615 | 272.0685 | 272.0685 | -0.03 | **271.0612** | 1.49 ± 0.03 | 3.09 ± 0.23 | 10.11 ± 0.65 | 1.81 ± 0.03 |
| Flavones |  |  |  |  |  |  |  |  |  |  |  |
| Apigenin | 5.83 | C_15_ H_10_ O_5_ | 269.0453 | 270.0524 | 270.0528 | -1.65 | **269.0445** | <L.O.Q. | 2.67 ± 0.00 | 3.01 ± 0.00 | <L.O.Q. |
| Apigenin diglucoside | 2.03 | C_27_ H_30_ O_15_ | 593.1501 | 594.1570 | 594.1585 | -2.50 | **593.1586**; 547.1098; 353.0726 | N.D. | N.D. | <L.O.Q. | N.D. |
| Luteolin | 5.30 | C_15_ H_10_ O_6_ | 285.0404 | 286.0477 | 286.0477 | -0.01 | **285.0405**; 241.0522; 133.0270 | N.D. | N.D. | 3.05 ± 0.01 | <L.O.Q. |
| Luteolin-7-O-apiosyl-glucoside | 3.95 | C_26_ H_28_ O_15_ | 579.1369 | 580.1441 | 580.1428 | 2.16 | **579.1355** | N.D. | N.D. | 2.98 ± 0.00 | <L.O.Q. |
| Coumarins |  |  |  |  |  |  |  |  |  |  |  |
| Esculetin | 3.71 | C_9_ H_6_ O_4_ | 177.0189 | 178.0265 | 178.0266 | -0.30 | **177.0190**; 133.0315 | ID | ID | ID | N.D. |
| Esculetin-6-O-glucoside | 2.87 | C_15_ H_16_ O_9_ | 399.0717 | 340.0787 | 340.0794 | -1.91 | 339.0744; 296.1057; **192.0049** | N.D. | N.D. | ID | ID |

Results are expressed in mg/g of extract. Results express mean ± standard deviation. L.O.Q.= limit of quantification, ID= identified compound, N.D.= Not detected compound.

Table Supplementary 2. Linearity information of calibration curves.

| **Composites** | **m** | **b** | **r** | **L.O.D. (mg/mL)** | **L.O.Q. (mg/mL)** |
| --- | --- | --- | --- | --- | --- |
| **Hydroxybenzoic acids** |  |  |  |  |  |
| Gallic acid | 100000000 | -127541.0 | 0.9939 | 0.0017 | 0.0025 |
| Protocatechuic acid | 70000000 | -57925.0 | 0.9994 | 0.0014 | 0.0026 |
| Syringic acid | 9000000 | 3625.2 | 0.9990 | 0.0038 | 0.0135 |
| Ellagic acid | 30000000 | -69214.0 | 0.9987 | 0.0036 | 0.0065 |
| **Hydroxycinnamic acids** |  |  |  |  |  |
| Caffeic acid | 100000000 | -6722.4 | 0.9982 | 0.0004 | 0.0013 |
| Chlorogenic acid | 60000000 | -22815.0 | 0.9998 | 0.0010 | 0.0025 |
| **Stilbenes** |  |  |  |  |  |
| Resveratrol | 200000000 | -827829.0 | 0.9865 | 0.0043 | 0.0048 |
| **Flavones** |  |  |  |  |  |
| Luteolin | 200000000 | -2000000.0 | 0.9933 | 0.0102 | 0.0106 |
| **Flavanones** |  |  |  |  |  |
| Hesperetin | 300000000 | -641511.0 | 0.9973 | 0.0023 | 0.0026 |
| Naringenin | 2000000 | -1808.4 | 0.9997 | 0.0197 | 0.0635 |
| **Flavanols** |  |  |  |  |  |
| Myricetin | 100000000 | -1000000.0 | 0.9969 | 0.0104 | 0.0113 |
| Quercetin | 90000000 | -1000000.0 | 0.9925 | 0.0115 | 0.0125 |
| Rutin | 200000000 | -190659.0 | 0.9980 | 0.0011 | 0.0016 |
| **Flavan-3-ols** |  |  |  |  |  |
| Catechin | 100000000 | -125463.0 | 0.9983 | 0.0016 | 0.0025 |
| Epicatechin | 200000000 | -104706.0 | 0.9992 | 0.0007 | 0.0011 |
| Epicatechin gallate | 90000000 | -84240.0 | 0.9733 | 0.0014 | 0.0023 |
| Gallocatechin | 70000000 | -5420.9 | 0.9963 | 0.0006 | 0.0019 |
| Epigallocatechin | 100000000 | -16929.0 | 0.9993 | 0.0005 | 0.0014 |
| Procyanidin B1 | 100000000 | -74992.0 | 0.9990 | 0.0011 | 0.0020 |
| Procyanidin B2 | 100000000 | -61835.0 | 0.9984 | 0.0010 | 0.0019 |
| Procyanidin C1 | 70000000 | -46555.0 | 0.9987 | 0.0012 | 0.0025 |

m= slope; b= intercept; L.O.D.= limit of detection; L.O.Q.= Limit of quantification. L.O.D and L.O.Q. were calculated with the signal-to-noise ratio (S/N).


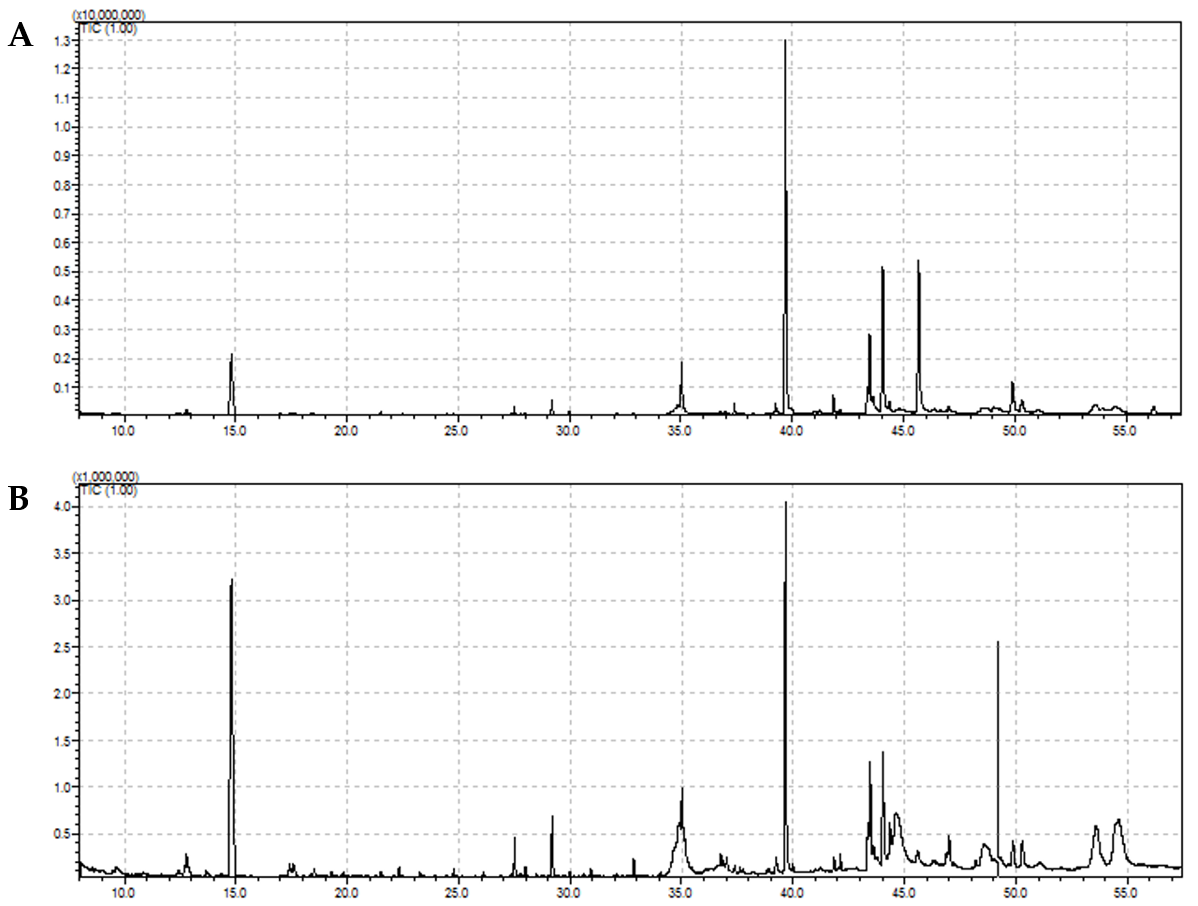


Figure S1. Chromatogram of the lipid profile of the non-polar extracts of vineyard pruning. A) Chromatogram of toluene extract of vineyard pruning extracts in GC-MS. B) Chromatogram of chloroform extract of vineyard pruning extracts in GC-MS.
